# Supplementary material for: Melittin Tryptophan Substitution with a Fluorescent Amino Acid Reveals the Structural Basis of Selective Antitumor Effect and Subcellular Localization in Tumor Cells
Source: Toxins (Basel). 2022 Jun 22;14(7):428. doi: 10.3390/toxins14070428 (PMC9318513; doi:10.3390/toxins14070428)
Supplement: Supplementary file 1 [file toxins-14-00428-s001.zip › toxins-1698414-supplementary.pdf]

toxins

MDPI

Supplementary Materials

Melittin Tryptophan Substitution with a Fluorescent Amino Acid Reveals the Structural Basis of Selective Antitumor Effect and Subcellular Localization in Tumor Cells

Yonghui Lv, Xu Chen, Zhidong Chen, Zhanjun Shang, Yongxiao Li, Wanting Xu, Yuan Mo Xinpei Wang, Daiyun Xu, Shengbin Li, Zhe Wang, Meiying Wu and Junqing Wang

Figure S1. (a) Mass spectra analyse of Melittin. MS characterization: M.W. = 2846.53 Da; found: 2846.73 Da. **(b)** Identifications of peaks in the HPLC chromatogram. The Melittin, which eluted with 1 mL/min, injection volume: 10 μL, detection wavelength: 220 nm, purity: 99.03%.

a

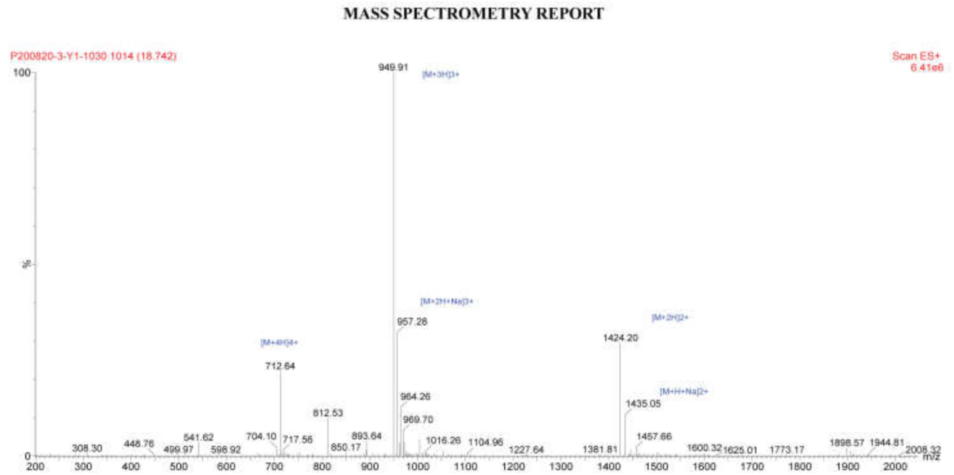

b

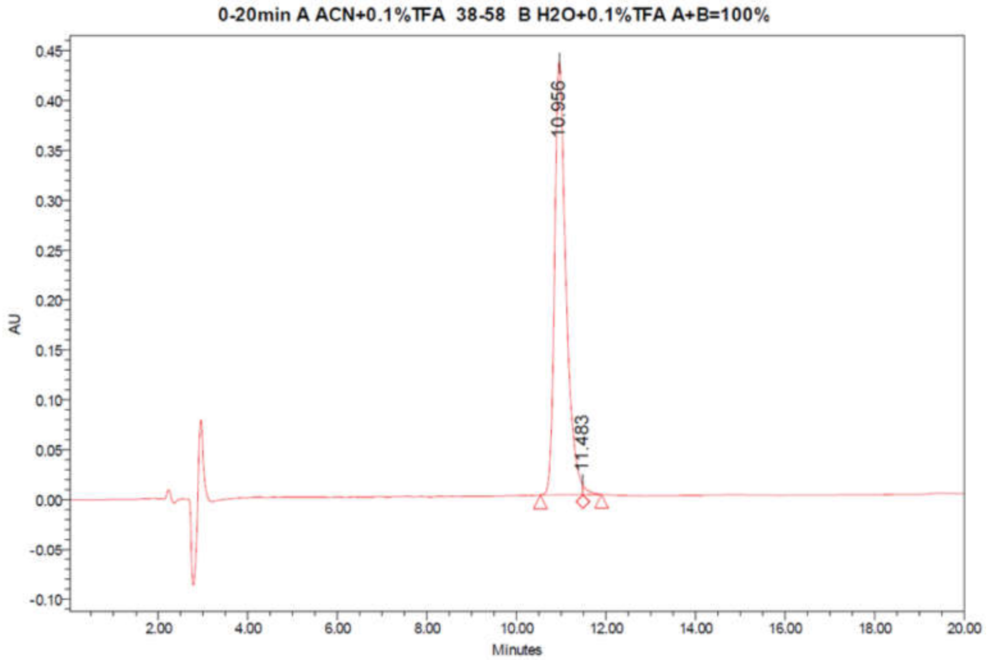

Processed Channel: 2487Channel 1

	Processed Channel	Retention Time (min)	Area	% Area	Height
1	2487Channel 1	10.956	7689073	99.03	434531
2	2487Channel 1	11.483	75393	0.97	9670

Figure S2. (a) Mass spectra analyse of MEL^{FL}. MS characterization: M.W. = 2962 Da; found: 2961.2 Da. **(b)** Identifications of peaks in the HPLC chromatogram of MEL^{FL}, which eluted with 1 mL/min, injection volume: 10 μL, detection wavelength: 220 nm, purity: 99.62%.

a

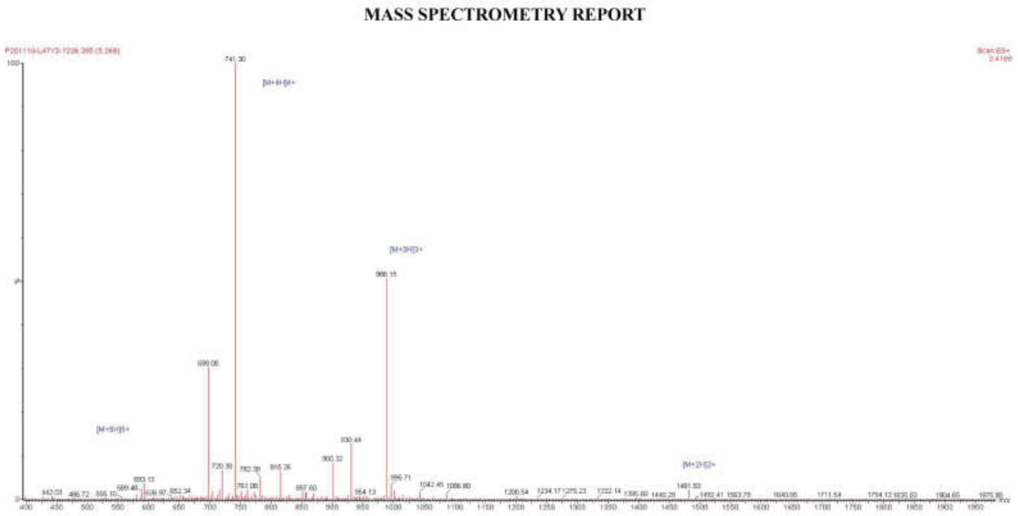

b

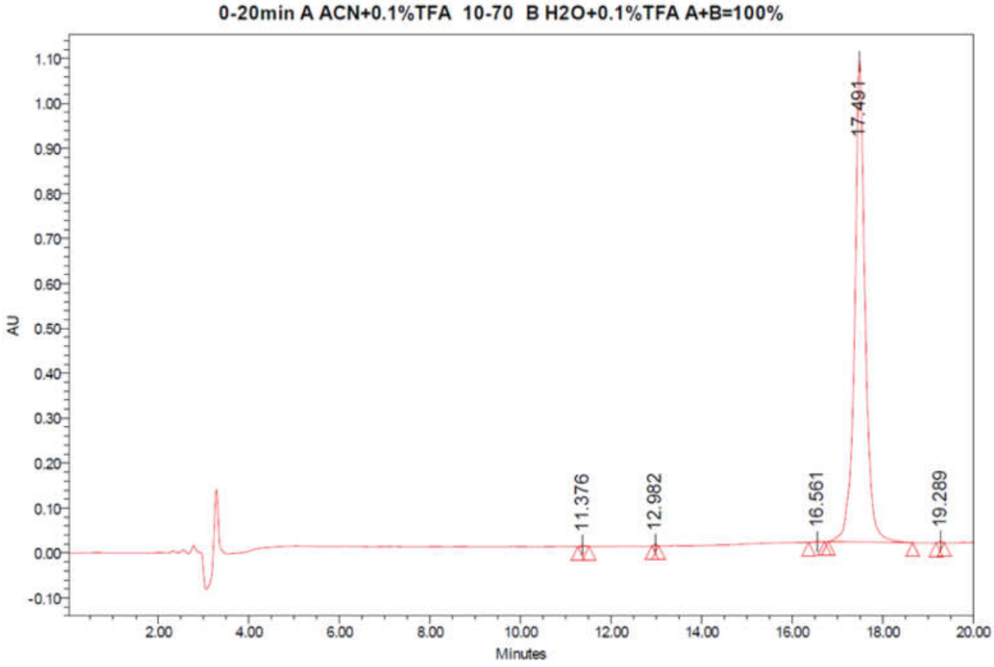

Processed Channel: 2487Channel 1

	Processed Channel	Retention Time (min)	Area	% Area	Height
1	2487Channel 1	11.376	19247	0.11	2701
2	2487Channel 1	12.982	16694	0.10	3681
3	2487Channel 1	16.561	13451	0.08	1404
4	2487Channel 1	17.491	17359089	99.62	1074723
5	2487Channel 1	19.289	16245	0.09	3280
